# Cuttlefish: Color Mapping for Dynamic Multi‐Scale Visualizations

**DOI:** 10.1111/cgf.13611

**Published:** 2019-03-26

**Authors:** N. Waldin, M. Waldner, M. Le Muzic, E. Gröller, D. S. Goodsell, L. Autin, A. J. Olson, I. Viola

**Affiliations:** ^1^ TU Wien Vienna Austria; ^2^ VRVis Vienna Austria; ^3^ The Scripps Research Institute La Jolla CA USA; ^4^ King Abdullah University of Science and Technology (KAUST) Saudi Arabia

**Keywords:** multiscale visualization, illustrative visualization, molecular visualization, I.3.3 [Computer Graphics]: Picture/Image Generation, I.3.7 [Computer Graphics]: Three‐dimensional Graphics and Realism; Color, Shading, Shadowing, and Texture

## Abstract

Visualizations of hierarchical data can often be explored interactively. For example, in geographic visualization, there are continents, which can be subdivided into countries, states, counties and cities. Similarly, in models of viruses or bacteria at the highest level are the compartments, and below that are macromolecules, secondary structures (such as α‐helices), amino‐acids, and on the finest level atoms. Distinguishing between items can be assisted through the use of color at all levels. However, currently, there are no hierarchical and adaptive color mapping techniques for very large multi‐scale visualizations that can be explored interactively. We present a novel, multi‐scale, color‐mapping technique for adaptively adjusting the color scheme to the current view and scale. Color is treated as a resource and is smoothly redistributed. The distribution adjusts to the scale of the currently observed detail and maximizes the color range utilization given current viewing requirements. Thus, we ensure that the user is able to distinguish items on any level, even if the color is not constant for a particular feature. The coloring technique is demonstrated for a political map and a mesoscale structural model of HIV. The technique has been tested by users with expertise in structural biology and was overall well received.

## Graphical abstract

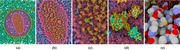

## 1 Introduction

**Figure 1:**
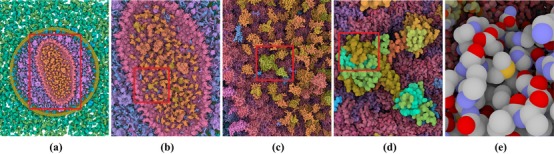
Interactive multi‐scale visualization of HIV using dynamic, categorical color coding for proteins and atoms: Zooming from an overview of the entire virus (a) to the capsid (b), the capsid interior (c), a single integrase protein in the capsid (d) and its atomic structure (e). Protein colors become more differentiated as we zoom in (a and b), and protein domains (c), secondary structures (d) and individual atoms (e) are progressively revealed through dynamic color adaptations. The red box indicates the zoom‐in location.

With improving technology, data sets and models are becoming larger and more complex. Furthermore, they are often available on multiple levels of detail. For example, it is possible to visualize an entire human immunodeficiency virus (HIV) [LMAPV15] from the compartment to the atomic level with its complete macromolecular composition. Multiple spatial magnifications need to be traversed with the camera to obtain a comprehensive understanding of the overall structure. An exploration can start at the compartment level, continue on a scale where single proteins are recognizable, zoom further into their domains and secondary structures, all the way to the amino acids and atoms (Figure 1). Similarly, geographic information is often conveyed on multiple scales, such as quantitative data that are aggregated from a district level up to a continent level [STH03]. Another example for dynamic multi‐scale visualizations are zoomable treemaps [BL07] and other types of hierarchically aggregated visualizations [EF10].

**Figure 2:**
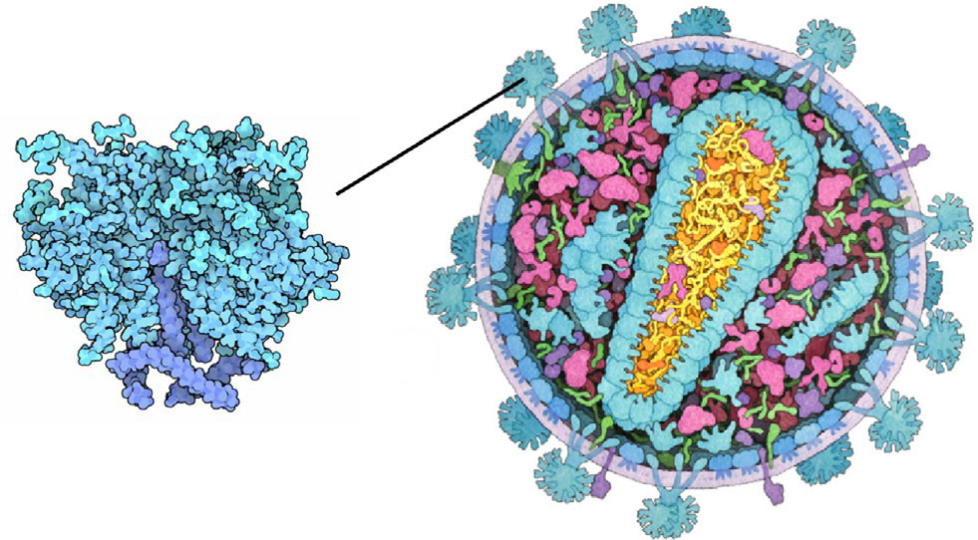
Multi‐scale illustration of HIV [RCS11]: overview of the virus on the right and close‐up on the envelope protein on the left. In the close‐up, different shades of blue are used to distinguish between protein domains, and carbon atoms are slightly darker.

**Figure 3:**
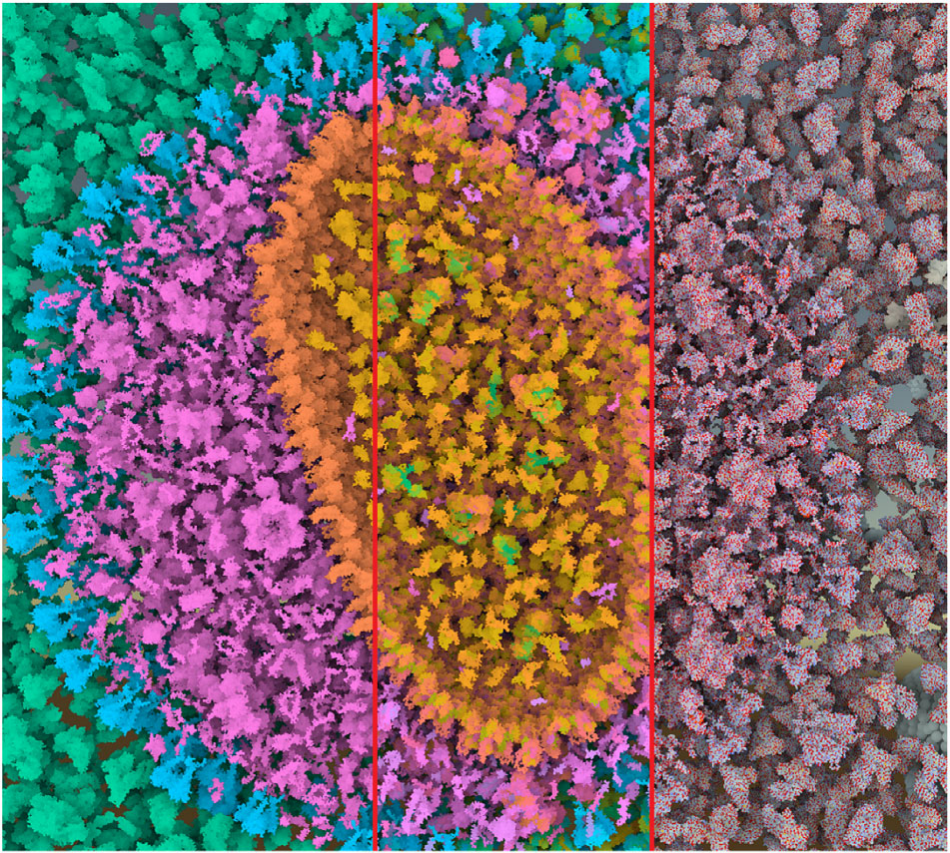
Comparison image of HIV with coloring based on compartments (left), secondary structures (middle) and atoms (right).

The color mapping of the data needs to take these different levels into account. Currently, illustrators carefully select color assignments for each detail level of their illustrations. In Figure 2, an illustrator depicts different protein types by color in the overview on the right. In the close‐up on the left, he uses different shades of blue to distinguish between the protein domains, which are not indicated in the overview. In interactively zoomable visualizations, a static, single‐scale color assignment will either lead to a loss or excess of information. If the coloring depends on the details, the result will be prone to salt‐and‐pepper noise (Figure 3 right), while a high‐level coloring will remove detail information (Figure 3 left).

A naive approach to multi‐scale color mapping independently defines colors for each zoom level and then blends the colors while zooming. However, several issues can occur. First, with independent color assignments for each zoom level, colors may change significantly when zooming. This can cause disorientation during zooming due to considerable color changes and may also cause artificial hues or greyed out colors when blending between levels [CWM09]. Second, there is a loss of hierarchical information when transitioning from one level to the next. For example, when looking at two neighbouring compartments in Figure 1(a) and zooming in, it may be hard to remember which compartment certain proteins belong to if their colors are assigned independently of their respective compartments. To take the data hierarchy into account, color spaces for hierarchical visualizations have been proposed in the past [FWR99, TdJ14]. These approaches are targeted towards static visualizations and therefore assign unique hues to each child node in a tree. This leads to poor scalability with respect to color discriminability. In hierarchical data structures, the number of items per level grows exponentially. This scalability issue can be resolved by exploiting the interactive navigation capabilities – and thereby induced dynamic visibility changes – of dynamic multi‐scale visualizations.

We propose a semantic color zooming method for multi‐scale visualizations, based on a view‐dependent, hierarchical color scheme. Starting from the highest hierarchy level, we progressively free up unused color space and redistribute it to visible items defined by the current zoom level and item visibility. This allows the user to distinguish items at different levels while maintaining group coherency. Our contributions are:

1. A hue assignment that dynamically adapts both to the current viewpoint and hierarchical structure of the data, allowing the user to distinguish between groups of items and items within a group.
2. Two methods that use luminance to enhance the color mapping while maintaining maximum saturation for the coloring method. The first method encodes additional quantitative information. The second method encodes structural properties across multiple zoom levels.

A small expert usability study was conducted. It shows that participants can distinguish biological structures on multiple scales through dynamic multi‐scale color mapping, while rating the visualization as highly appealing. The usability study also indicates that there was no confusion caused by the dynamically changing colors. This was a potential cause for concern, as it is technically possible for the colors to change significantly between perspectives. The focus of this paper is on the creation of a dynamic hierarchical coloring scheme. Interactive multi‐scale exploration of data is increasing in prevalence and importance. Dynamically reassigning color and other visual channels is likely to attract increased research efforts. Dynamic coloring is based on previously researched methods, and the usability study is intended to verify if the extension into multi‐scale visualization is useful and does not cause confusion.

## 2 Related Work

The selection of a color map for data depends heavily on the visualization goal. Clear visibility and also harmonious and appealing colors are important [Iha03], as well as the assignment and mixing of colors [WGM*08]. There are many issues to consider, and as a result, guidelines have been published on how to decide which color scheme is most appropriate [BRT95, Hea96]. Our method uses a hue wheel, but expands it to be dynamic and includes the hierarchical structure of the data in order to create a seamless yet expressive multi‐scale visualization. The items within a group use analogous colors, which is similar to the use of an analogous hue wheel. Hue wheels have been used before, and a good overview of research in this direction, types of hue wheels, color schemes, and color harmony is given in the books by Stone [Sto03] and Rhyne [Rhy16]. Interesting research in the field of color harmony has been published by Cohen‐Or *et al*. [COSG*06], Lu *et al*. [LKPL14] and Wijffelaars *et al*. [WVW*08]. Wijffelaars *et al*. employed curves, e.g. Bezier curves, which allow the user the intuitive specification of the parameters of the color palette. Cohen‐Or *et al*. [COSG*06] describe a method for changing the colors of an image in order to create a more harmonious picture. A hue wheel is split up into multiple sectors that are harmonious with each other. The size and number of the sectors can vary. The colors of the image are then altered to be within the different sectors. Lu *et al*. [LKPL14] approached color harmony from a machine learning perspective. They trained their programme on images that were ranked based on color harmony. Their prediction of the harmony of a new picture outperforms several rules from color harmony. While a discussion of color harmony lies outside of the scope of this paper, an investigation into this could be beneficial for coloring neighbouring structures or other elements on the same hierarchical level with the coloring method described in this paper. More information on color harmony can be found in the aforementioned papers.

There have also been guidelines for two‐dimensional color maps, such as by Bernard *et al*. [BSM*15], and the software ColorMap‐Explorer by Steiger *et al*. [SBT*15]. Furthermore, several tools have been developed to assist users to select appropriate colors for maps, such as Colorbrewer [HB03], and color maps for clusters of non‐hierarchical data, such as ‘i want hue' [Med16]. A simplistic clustering method can, however, cause perceptual issues, either due to the large number of clusters or their layout. In cases, such as colored maps, visually dominant items may perceptually suppress small groups or areas [LSS13]. Visualization of information as given for maps can lead to small areas being next to much larger ones. In this case, it may be difficult to spot the small regions. Lowering the saturation of the larger areas allows the small ones to stand out more clearly. Perception can also be affected by contrast effects, which may need to be dealt with [MSK14]. Particular care needs to be taken when dealing with higher dimensional data [MBS*14], so that the distances between the clusters do not become too distorted.

While there has been intense research on color mapping in visualization in the past, there are only few color mapping techniques for multi‐scale visualizations. Fua *et al*. [FWR99], for instance, introduce proximity‐based coloring for hierarchical parallel coordinates. Polylines belonging to the same cluster are assigned similar hues, while unused ‘hue buffers’ between clusters ensure that individual clusters can be clearly distinguished. Tree Colors [TdJ14] uses a tree‐based method to assign hues to hierarchical data structures. The approach uses the HCL space, a cylindrical coordinate version of the CIELab space with hue, chroma and luminance. The method assigns to each cluster a different hue. The root of the tree is depicted without saturation in grey, and the hue is divided into different wedges. A hue wedge is assigned to each tree branch, with some unused buffer space between branches. As the tree spreads out, the saturation of the nodes increases, and each level gets its own smaller wedge. Essentially, each level has its own hue ring with a defined chroma. These techniques were designed for static visualizations, where all colors are visible at the same time. Therefore, the number of possible colors is severely limited, leading to discriminability problems for very large data sets. In contrast, our method dynamically reassigns colors based on the current visibility. This increases distinguishability as the number of visible items decreases, e.g. because of zooming in. We also take care that the introduced color shifts of individual items are minimized.

Prior work on dynamic color mapping for multi‐scale molecular data has been proposed by Waldin *et al*. [WLW*16]. Their approach is somewhat limited because of the way coloring is done on lower levels of the hierarchy. The coloring method allows for color overlap between children of different parents. This requires visible separation in space, as otherwise it becomes difficult to distinguish between different items or groups. Such a scenario, however, might easily occur, like in hierarchical map data. In this work, we extend the dynamic color mapping approach by Waldin *et al*. to be applicable beyond the biology domain. Color overlap can be prevented. This allows it to be applied to any hierarchical data. Furthermore, due to algorithmic optimization, the coloring can be calculated in one step. In contrast, the coloring by Waldin *et al*. [WLW*16] requires an ongoing, physically based simulation to occur in the background. In addition, we combine multi‐scale categorical color mapping with a hierarchical quantitative color scheme to convey hierarchically aggregated quantitative information. We demonstrate this aggregation for molecular visualization and geographic map visualization.

## 3 Overview

Our goal is to propose an effective dynamic color mapping technique. The technique enhances the user's ability to distinguish between items and groups on each zoom level. The items and groups to be distinguished change from zoom level to zoom level and additional information, such as domain knowledge, is necessary to identify them. Our method automatically adapts to the current viewpoint and fulfils the following requirements:

1. On each zoom level, the associated main items (i.e. those items that are best visible at the current level) should be clearly distinguishable from each other.
2. The inherent hierarchy of the data structure should be reflected in the color coding.
3. In continuously zoomable multi‐scale visualizations, the color transitions between the zoom levels should be smooth to avoid abrupt appearance changes or orientation loss.
4. The coloring must be coherent, i.e. a small shift in the view should not alter the colors substantially.
5. The coloring must be consistent, i.e. the color mapping from a specific view should always be the same.
6. The visualization should be aesthetically pleasing to engage a broad audience.

We employ the HCL color space to create a hierarchical coloring scheme. HCL (hue, chroma, luminance) is a cylindrical coordinate version of the perceptually uniform CIELab space. Using HCL allows us to determine the hue through one parameter, which is used in our method, while maintaining the usual benefits of a perceptually uniform color space. We use the *hue* to distinguish between items at the respective zoom levels, as described in Section 4. We describe the dynamic adaptation of the hues as the user is panning the scene to ensure color distinguishability even in cases with dozens of different entities. Furthermore, we describe the hierarchical subdivision of hue ranges as the user zooms in (Section 4). The approach is demonstrated on an HIV model in Section 4.3 and political maps in Section 4.4. *Luminance* encodes hierarchical aggregation of quantitative information from lower hierarchy levels (Section 5.1) or indicates items from lower hierarchy levels to smooth color transitions (Section 5.2). An interpolation method for a smooth color transition is described in Section 6. Finally, we demonstrate the effectiveness of our technique in a usability study with professionals and students from the field of molecular biology (Section 7), where they visually explored a multi‐scale model of an HIV.

## 4 Dynamic Hierarchical Hue Assignment

**Figure 4:**
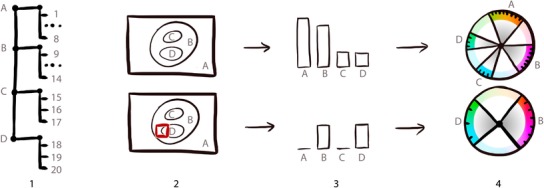
Overview of the view‐dependent hue assignment: (1) an exemplary two‐level hierarchy of four groups (A,B,C,D) and a total number of 20 different items: For the current viewport (2), visibility information is extracted (3) and the color assignment is updated (4).

The task is to find colors for visible items, so that users can distinguish between them, but also understand their hierarchical grouping. We use hue for this distinguishing and visual grouping of items, since it is recommended to use hue for low‐frequency information [BRT95]. The task of finding suitable hues can be considered as defining wedges on an iso‐luminant hue circle in HCL space, centred in the grey spot, as illustrated in Figure 4, fourth column. Each wedge corresponds to a group of items, and individual items are placed equidistantly within these wedges, as illustrated in Figure 4, top row. To explain the dynamic coloring scheme, we define the following terms:

1. $\alpha_{max}$: The maximum permitted angle between two neighbouring items on the hue circle (for item distinguishability).
2. $\beta_{max}$: The maximum permitted angle between two neighbouring wedges (for group distinguishability).
3. α: The angle between two neighbouring items inside a group on the hue circle. $\alpha \;=\;\min(s_{\alpha },\;\alpha_{max})$.
4. β: The angle between two neighbouring groups (wedges). $\beta \;=\;\min(s_{\beta },\;\beta_{max})$.
5. $s_{\alpha }$: A function for calculating α according to the number of visible items. It is referred to as ‘scaling function’ because it scales α based on the number of visible items.
6. $s_{\beta }$: A function for calculating β according to the number of visible groups. Referred to as ‘scaling function’ because it scales β based on the number of visible groups.
7. $h_{ij}$: The hue value of the visible item *j* in group *i*.
8. $H_{i}$: The hue value of the parent of group *i*.
9. *n*: The number of visible groups.
10. $m_{i}$: The number of items in group *i*.

**Figure 5:**
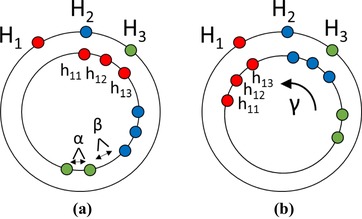
Placement of items on the hue circle. The parents are on the outer circle. The children are on the inner circle. The colors indicate which items belong together. $h_{ij}$ and $H_{i}$ are marked. (a) Shows the placement of the child items on the inner circle according to Equation (1). α and β are the angles between items and wedges, respectively. (b) Shows the position of the child items after the rotation by γ according to Equation (5).

Figure 5(a) illustrates α, β and different groups, parents and children.

$\alpha_{max}$ and $\beta_{max}$ are scalar values. They define the maximum color differences between neighbouring items and neighbouring groups, respectively. This is particularly important when dealing with few items or groups, as it can enforce similar colors within a group, rather than having few items spread out across a large section of the hue circle. $s_{\alpha }$ and $s_{\beta }$ are functions that allow the distribution of hues to scale with the number of visible items and groups. Using $\alpha_{max}$, $\beta_{max}$, $s_{\alpha }$ and $s_{\beta }$, we can calculate α and β. With this, we calculate $h_{ij}$:

(1) $$h_{ij}=\left(i-1\right)\cdot \beta +\left(\sum_{k=1}^{i-1}m_{k}\right)\cdot \alpha +\left(j-1\right)\cdot \alpha ,$$

where $m_{k}$ is the number of visible items in group *k*. Using Equation (1) is the same as placing the items in order around the hue circle with distance α between items of the same group and β between different groups. An illustration showing the placement according to Equation (1) can be seen in Figure 5(a).

The visualization technique must be able to adapt the coloring to the data and viewpoint. In order to achieve this, $\alpha_{max}$ and $\beta_{max}$ are changeable parameters. For example, the user may want all items within a group to have the same color, so he can easily see the distribution of groups. In this case, he sets $\alpha_{max}$ to 0. In general, if $\beta_{max}$ is large and $\alpha_{max}$ small, then the emphasis is on which group on item belongs to. If $\alpha_{max}$ is large, then the emphasis is on distinguishing items within a group.

$s_{\alpha }$ and $s_{\beta }$ are used for adapting α and β to the number of visible items and groups. While these are not user parameters, they can be adapted to the data set. For example, the user may want to have the items easily distinguishable when there are few visible groups, but emphasize group belonging when there are many visible groups.

Defining $\alpha_{max}$, $\beta_{max}$, $s_{\alpha }$ and $s_{\beta }$ like this allows for multiple coloring strategies to be available. In this paper, we have chosen $s_{\beta }$ (Equation (2), *n* being the number of visible groups) in such a way, that if $s_{\beta }$ is less than $\beta_{max}$, then all items belonging to the same group have the same color. If this is not the case, then the items spread out as much as possible, as long as α is less than $\alpha_{max}$. Our $s_{\alpha }$ is shown in Equation (3), with an elaboration for a data set immediately below:

(2) $$s_{\beta }=\frac{360^{\circ }}{n},$$

(3) $$s_{\alpha }=\frac{360^{\circ }-n\cdot \beta }{\sum_{i=1}^{n}\left(m_{i}-1\right)}.$$

This leads to three possible cases. In the first case, α and β are equal to ${\mathrm{alpha}}_{max}$ and $\beta_{max}$, respectively. In the second case, α is equal to $s_{\alpha }$ in Equation (3), and β is equal to $\beta_{max}$. In the third case, α is zero and β is equal to $s_{\beta }$ in Equation (2).

**Figure 6:**
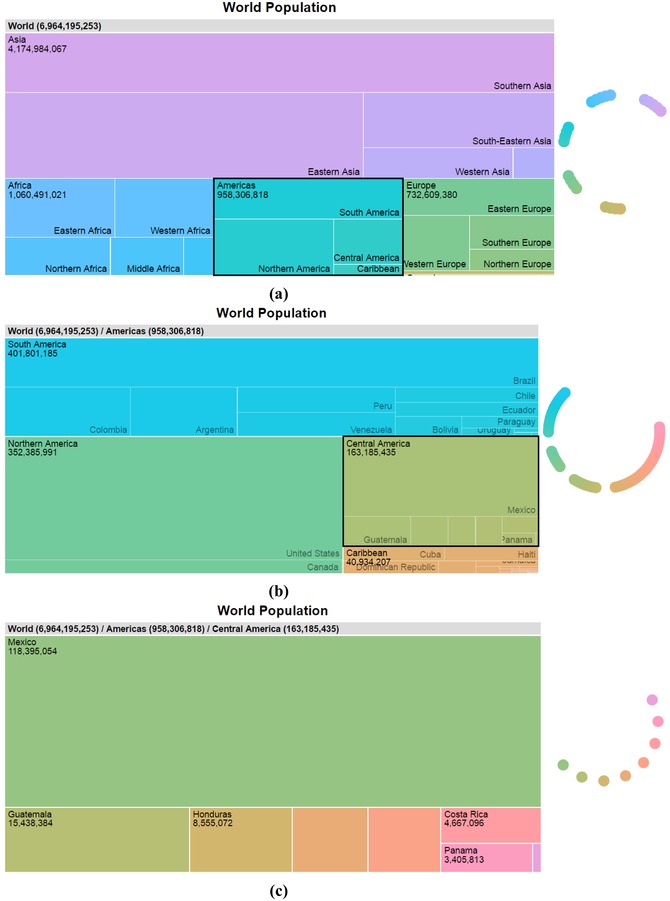
Applying Cuttlefish on a zoomable treemap [Gan16]: (a) The initial world view is zoomed to (b) the Americas and (c) Central America. In this example, we use an $\alpha_{max}$ of 5° between items, a $\beta_{max}$ of 30° between wedges and the countries’ areas (encoded by size) as weights to balance the hue shift across scales. The color wheels to the right of the tree maps show the assigned colors.

In Figure 6, we can see multiple viewpoint levels (world view, Americas, Central America). In Figure 6(a), the emphasis is placed on group belonging, not individual items. Of course, the scaling functions can be modified to emphasize groupings as desired. In the treemap example shown in Figure 6(c), the number of items and the chosen maximum hue angles are sufficiently small so that α and β are equal to $\alpha_{max}$ and $\beta_{max}$, respectively.

When choosing $\alpha_{max}$, $\beta_{max}$, $s_{\alpha }$ and $s_{\beta }$, it is important that α remains smaller than β. Otherwise, the distance between groups is smaller than the distance between items within a group. This can lead to confusion between items from different groups, as well as a loss of hierarchical continuity in the color coding (requirement 2).

However, if there are a lot of visible items and groups, colors can become very similar and hard to distinguish, such as in Figure 1(a). This effect is actually desirable to avoid salt and pepper noise. However, as we zoom in, we want to increase distinguishability (requirement 1), while maintaining information about the hierarchy. We therefore introduce a hierarchical view‐dependent color adaptation approach that takes the number of visible items into account, and thereby adjusts the scale functions $s_{\alpha }$ and $s_{\beta }$ dynamically, based on the current visibility, while minimizing the difference in hue between child and parent items.

### 4.1 Hierarchical view‐dependent color adjustment

As the user zooms in and explores the visualization, many items may not actually be visible in the current view, as illustrated in the lower row of Figure 4. In this configuration, the available color space becomes underexploited. We therefore recompute and optimize the color assignment on‐the‐fly as items and groups appear and disappear. An overview of the mechanism is illustrated in Figure 4.

For every zoom or pan operation by the user, the hues of the ordered set of visible items are reassigned (Equation (1)), and the scaling functions are re‐computed. We explain this with $H_{i}$ – the hue of the parent of group *i*. To avoid unnecessarily large color shifts (requirement 2) while navigating, we want to minimize the distances between $H_{i}$ and $h_{ij}$. In order to achieve this, we introduce a rotational shift γ, i.e. the position of an item on the hue circle becomes $h_{ij}+\gamma$, where $h_{ij}$ is defined through Equation (1). This means that the distance between the position of an item on the hue circle and $H_{i}$ is equal to $|H_{i}\;-\;h_{ij}\;-\;\gamma |$. To find γ, we minimize the sum of squared distances, which we define as *G*. We allow the distances to be weighted, in case the user wants the color of a specific section to be highly consistent between levels. This means *G* is:

(4) $$G=\sum_{i=1}^{n}\sum_{j=1}^{m_{i}}\omega_{ij}{\left(H_{i}-h_{\mathrm{ij}}-\gamma \right)}^{2},$$

where $\omega_{ij}$ are weight factors that can be used to put more emphasis on large items or groups, so that their colors remain more stable than others. An illustration of the shift by γ can be seen in Figure 5.

For the sake of clarity, we maintain the equidistant hue distribution of items given by α and β, i.e. the distances between items and groups, respectively. Therefore, we apply a simple global rotation (similar to a rigid transformation) instead of shifting individual wedges or items independently. This means *G* is minimal if γ is minimized according to the average weighted angular displacement between each visible item's currently assigned hue $h_{ij}$ and its parent $H_{i}$. Finding the minimum by taking the derivative of *G* (Equation (4)) with respect to γ, setting the derivative equal to zero and solving for γ lead to the following result:

(5) $$\gamma =\frac{\sum_{i}\sum_{j}\left(\left(H_{i}-h_{\mathrm{ij}}\right)\cdot \omega_{\mathrm{ij}}\right)}{\sum_{i}\sum_{j}\omega_{ij}}.$$

The large weights reduce the color changes for important objects, as their large influence on γ will minimize their color shift. For instance, in Figure 6, the geographic area of the items was used as weight factor, which is also encoded by the size of the rectangles in the tree map. As the user zooms from a world view (Figure 6a) down to the Central American countries (Figure 6c), the weighted color shift ensures that Mexico, as the largest Central American country, maintains a similar color to its parent (Central America in Figure 6b). Smaller countries, like Panama, have little effect on γ and can have a large difference in color to its parent. Another example of this coloring technique can be found in Section 4.4.

Our continuous solution guarantees that a small change in visible items will lead to a small change in the coloring (requirement 4). The hue assignment calculation is deterministic, therefore the coloring is consistent and particular views always have the same color assignments (requirement 5). There are no default values listed here for $\alpha_{max}$, $\beta_{max}$ or other values, as they they are determined by what the user is interested in. However, we recommend the user start by having a large β first. This will allow him to get a good overview of the overall data set structure. The high contrast between the groups will aid the user in determining which groups are where. Afterwards, by increasing α, e.g. through a small $\beta_{max}$, the user can focus more on the content of the data at a level.

### 4.2 Hierarchical colors with overlap

The method from Section 4.1 prohibits color overlap between groups. This is important if items from different groups are spatially next to each other. If this is not the case, it is possible to allow for an overlap. Color mapping with color overlap is explained in this section.

**Figure 7:**
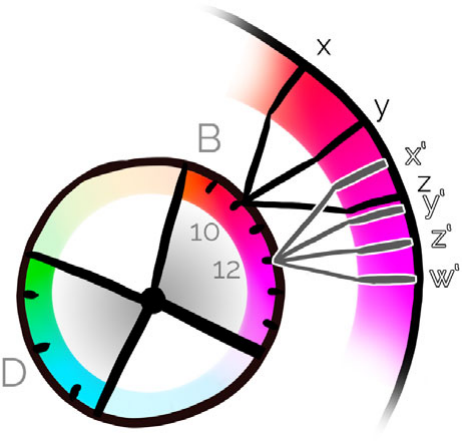
Hierarchical hue assignment by setting child wedges centred at the parent hue.

**Figure 8:**
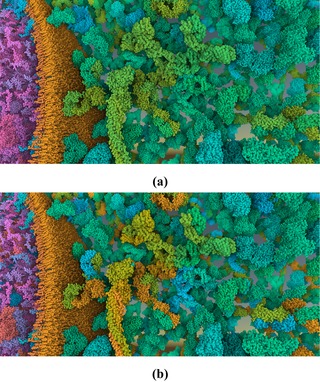
Effect of different $\alpha_{max}$: (a) 60° and (b) 180°. Mind how the domains of different proteins are assigned identical colors in (b).

When allowing for overlap, colors for each group of items are assigned independently. Given a parent hue of the *i* ^*th*^ group $H_{i}$, the hue ring is further sub‐divided into wedges centred around the parent hue. This is illustrated in Figure 7. The hue angle between the items is $\alpha_{max}$, by default, and can be scaled down so that all items fit within a pre‐defined maximum wedge size. Mind that hue wedges can overlap in this case, as $\beta_{max}$ and β are effectively ignored at lower levels of the hierarchy. This means that items from different groups can be assigned identical hues. While this supports distinguishability within groups and leads to minimal hue displacements, it may be difficult to distinguish groups from each other. We therefore only use this wedge sub‐division approach for hierarchical data where groups are clearly separated through other visual means, e.g. spatial separation or visual distinction. An example is shown in Figure 8.

Allowing color overlap has both advantages and disadvantages. Not allowing overlap is important if the groups are not separated significantly in space, as in the case of zoomable political maps in Section 4.4. If the items are visibly separable, as with the HIV example in Section 4.3, then the color overlap allows for larger distinguishability between items within a group. Of course, this can cause confusion if the groups are very close to each other. Which should be applied depends on how easily groups can be (spatially) separated without color.

### 4.3 Application: HIV

The coloring method with overlap from Section 4.2 is applied to an HIV data set containing multiple levels. The rendering method uses a spacefilling representation. See Kozlikova *et al*. [KKL*15] for an overview of different rendering methods of molecular data.

In Figure 1, the different levels of an HIV model are shown. When zooming into the HIV model, we start at the compartment level (1a), continue through the protein level (1b), to the domain (1c), secondary structure (1d) and atomic level (1e). A domain of a protein is a part of the protein sequence and is often individually stable and can often be folded. A domain contains secondary structures, such as α‐helices and β‐sheets. By applying the coloring method described in Section 4.2, the individual structures are distinguishable at each level. Furthermore, the similarity of colors within a group helps to indicate where one group begins and another ends. For instance, in Figure 1(b), individual proteins are assigned slightly different colors, while the hierarchical grouping (capsid hull and interior) remains visible. At the lowest hierarchy level, we use the pre‐defined CPK (Corey–Pauling–Koltun) color scheme for atoms to support domain knowledge (Figure 1e).

To determine the hue assignment, we extract visibility information by analysing the previously rendered frame. For each protein group *i*, we determine the number of visible protein types $m_{i}$. A protein type is visible, if at least one screen pixel is occupied by a protein of type *j*. We leverage GPU computing in a post‐processing operation, in order to compute the visibility of each protein type efficiently. Upon rendering, we generate an additional offline texture which contains, for each pixel, the unique identifier of the rendered protein. There are two GPU buffers which will, for each protein type, store the occupied pixel count and the total number of visible proteins, respectively. Subsequently, in a dedicated compute shader, we iterate through all the pixels, and we increment the pixel counter corresponding to the protein type stored in the video memory. At the same time, we also flag in a third dedicated GPU buffer the proteins whose unique IDs are present in the generated texture. This information will allow us to determine the number of visible instances for each protein type. In a second pass and in another dedicated compute shader, we then iterate through all the protein instances. For each protein which was flagged as visible in the previous pass, we increase the number of visible instances corresponding to the protein type. Since the computation is done in parallel, it is important to use atomic intrinsic operations to increment the counters, in order to avoid concurrent access to the same value from two different threads.

### 4.4 Application: zoomable political maps

**Figure 9:**
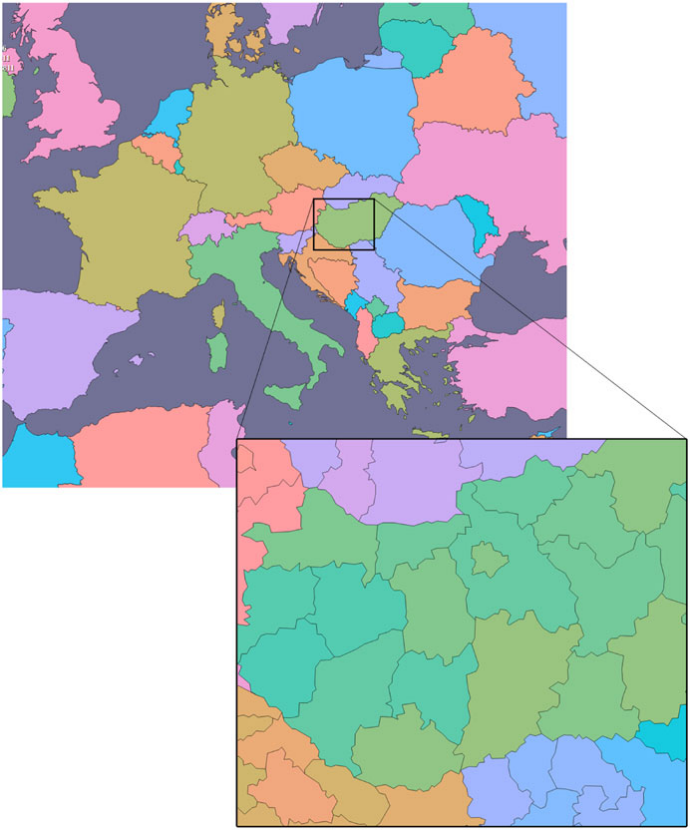
Zoomable political map: zooming in from Europe to Hungary.

The non‐overlap coloring method from Section 4.1 can be used for geographic visualization. Political maps use categorical colors to distinguish individual countries, states or districts. In Figure 9, we show a zoomable map with dynamic multi‐scale colors. Hues are initially assigned from an overview perspective of a map of Europe. As the user zooms in and countries are clipped from the view, the country colors are re‐adjusted for better distinguishability. At a pre‐defined zoom level, the map switches from a country to a state representation. The Cuttlefish algorithm takes care that each state is assigned a unique color, and that the hue shifts with respect to the parent (i.e. country) are minimized. This allows the grouping of states into countries to be visible despite lacking thicker borders at the country level.

## 5 Luminance Modulation

The dynamic hierarchical hue assignment described in Section 4 only affects the hue. This leaves the other two HCL channels available to encode additional information. We show two methods how to encode additional information using the luminance channel: to additionally encode hierarchically aggregated quantitative information (Section 5.1) and to subtly indicate items from a lower hierarchy level (Section 5.2). Chroma is affected by the luminance modulation and is not used for encoding information.

### 5.1 Quantitative luminance coding

**Figure 10:**
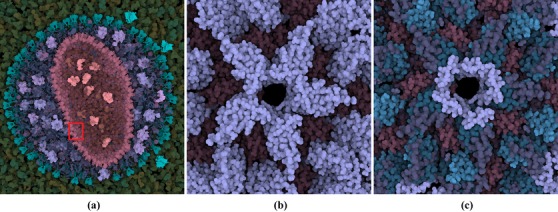
Use of luminance to indicate the presence of the lysine amino acid. A higher luminance indicates a higher number of amino acids. In (a), the presence in the proteins is shown. In (b) and (c), the concentration of lysine in a pentamer of the capsid is shown on the domain and secondary structure level, respectively. The red box in (a) indicates the location of (b) and (c).

When viewing the data at a high level, the user may be interested only in a subset with certain characteristics present on a lower level, such as the quantity of a type of amino acid in different molecular structures. Therefore, it may be necessary to propagate the information up to the current level through a hierarchical aggregation of quantitative information defined on a lower level. While luminance is a viable channel to encode aggregated quantitative information, there are drawbacks. An important step in understanding contrast between foreground and background was made by Whittle [Whi86] and Kingdom *et al*. [KW96]. A more thorough overview of luminance perception can be found in the article by Kingdom from 2011 [Kin11]. Essentially, the perception of a luminance value can be distorted, making an accurate comparison between minor differences in luminance difficult. Compensating these distortions has been investigated. An initial iterative approach for color was done by Mittelstädt *et al*. in 2014 [MSK14], with a real‐time algorithm in 2015 [MK15]. The goal of the method in this paper is not to give a perfect mapping from luminance to quantity. Given the nature of the data sets, there can be large sections which the user would like to ignore. The method is designed to maintain the hue while indicating regions of interest. For example, in Figure 10, it is visible that a large number of proteins contain a small number of the lysine amino acid.

Quantitative information is defined on the lowest hierarchy level of the data, for instance by items associated with data available per district. For each zoom level, the number of items is aggregated per displayable unit, such as the number of items per country. For each hierarchy level, the range of aggregated items is then mapped to a luminance range, with a higher luminance representing a higher number of items per unit. In our examples, we use a luminance range of [33,73] (maximum luminance range in HCL is [0,100]).

However, the color saturation (or chroma) for different luminance values depends on the limits of the monitor's RGB space. As the luminance decreases, the chroma must be reduced, or the value would be outside of the monitor's gamut. We can achieve the highest possible chroma of around 41 for a luminance of around 75, assuming the monitor is calibrated to sRGB. With decreasing luminance, the chroma decreases. We therefore also linearly interpolate between a chroma range of [22,41] in our example, based on the item's associated quantitative value. This means the colors are chosen from a cone in HCL space. The bottom of the cone is at luminance 33 and has a radius (chroma) of 22. The top of the cone is at luminance 73 and has a radius of 41.

The usual problems when using luminance to estimate magnitude still remain, such as accuracy and contrast. However, this method allows for the use of luminance while maintaining the hierarchical coloring method.

#### 5.1.1 Application: lysine concentration in HIV

The lysine amino acid, which contains reactive ends to join two or more molecules, is often used as the target for chemical crosslinking agents. This can be used to study how different subunits are arranged relative to one another in higher order assemblies. Lysine is often the target for these crosslinkers, because there are often many of these on the surface of typical proteins. Indicating to the user the prevalence of these amino acids, as well as their position is therefore desirable.

The results of the luminance modulation for the HIV data set for different levels are visualized in Figure 10. Figure 10(a) shows that lysine is fairly evenly distributed among the virus proteins. When zooming in (Figure 10b), it becomes obvious that lysine is present in the capsid protein, but only within certain secondary structures (as visualized in Figure 10c).

#### 5.1.2 Application: choropleth map

For the second example, we extend the zoomable political map from Section 4.4 with quantitative information and thereby create a choropleth map. Classic choropleth maps encode quantitative information for discrete areas using a quantitative color scale, where the hue is usually static, and the luminance is varied linearly or logarithmically with the associated quantitative information.

**Figure 11:**
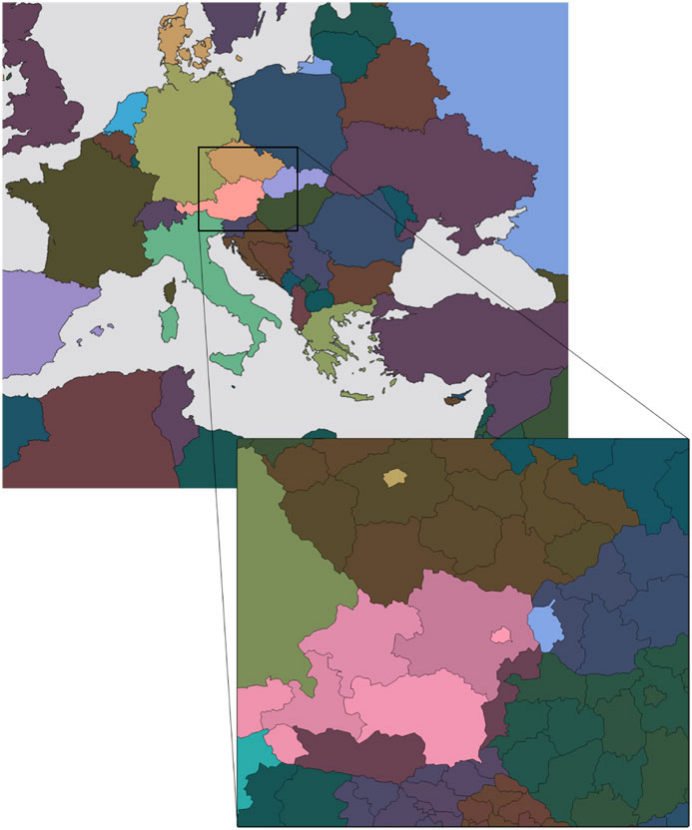
Zoomable choropleth map, where the hue encodes the political entity and luminance and chroma encode the number of publications produced by the depicted regions: zooming from Europe to Austria and its Eastern neighbours.

In our example, we use the political map in Figure 9 as the basis to start with. The hue is used to differentiate between the individual countries or states. We use luminance and chroma within the ranges described in Section 5.1 to encode publication number from a publication database of one of our collaborators. The publication counts are aggregated for each discrete zoom level from the publication records, where each publication record is associated with at least one affiliation and its geographic location, respectively. We linearly map the minimum and maximum publication number of the visible items to the luminance and chroma ranges so that publication activities can be compared between political regions on multiple scales. In Figure 11, for instance, it is noticeable that most publication output in this database is provided by central European countries, and in particular by the capital regions Vienna, Prague and Bratislava.

### 5.2 Luminance modulation of detail structures

Luminance can also be used to smooth the transition between levels by indicating lower level structures. For example, molecular illustrators sometimes use the luminance channel to indicate structural properties in lower hierarchy levels, as shown in the close up in Figure 2 or in the ‘Molecule of the Month’ example [Goo13]. Here, luminance is used to indicate structural information of lower levels. Hue is known to be an effective channel to encode low‐frequency information, while luminance is more effective for encoding high‐frequency information [BRT95]. Furthermore, encoding information from an adjacent hierarchy level can support orientation when seamlessly transitioning between zoom levels.

**Figure 12:**
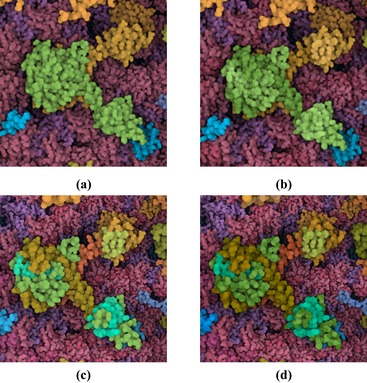
Comparison of domain level (top) and secondary structures level (bottom) without (left column) and with (right column) luminance modulation.

For each hierarchy level, we can therefore not only assign unique hues, but also indicate lower level structures with luminance values. These luminance values are unique within an item (but not between items) in our HIV application example. We set the base luminance to 73 and modulate this luminance value by up to 15, as illustrated in Figure 12. The modulation of protein secondary structures and atoms is shown in the top and bottom rows, respectively. For secondary structures, β‐sheets have lowered luminances and α‐helices increased luminances. Parts of the protein that contain neither are unchanged. In the case of atoms, the luminance is decreased if it is not a carbon atom (Figure 12d), creating an effect similar to the illustrator technique shown in Figure 2. With this small modulation, the luminance channel serves only as a subtle indication of lower level structural properties, to avoid extensive visual clutter and interference with shading cues.

## 6 Interpolating Between Hierarchy Levels

**Figure 13:**
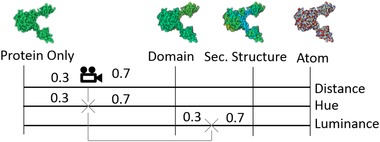
Interpolation of hue and luminance values for structural information in the HIV example (Section 4.3): world space distance to the camera defines the interpolation factor for hue and luminance offset (Section 5.2).

**Figure 14:**
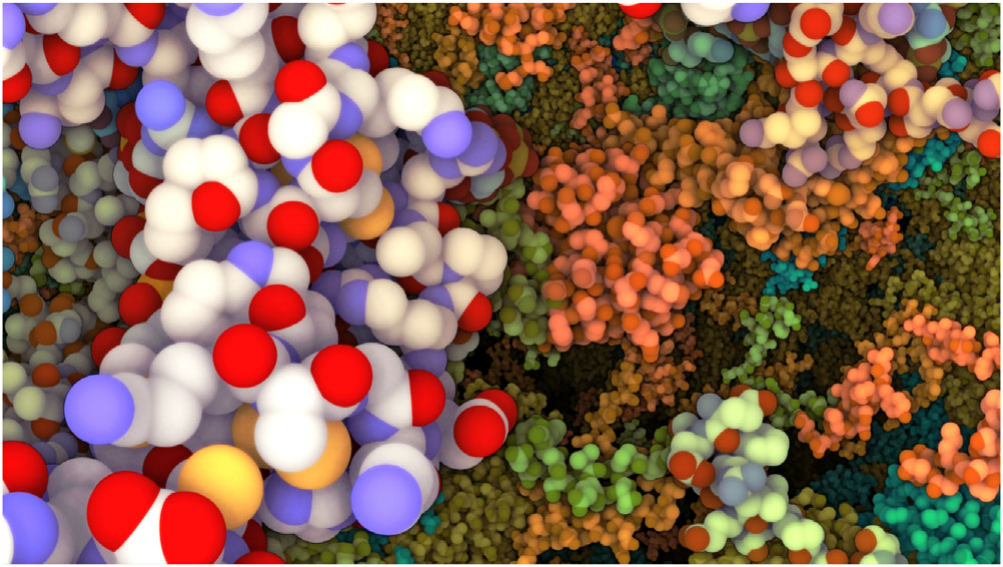
Multiple zoom levels are visible with dynamic coloring visible.

While zooming, the visualization may reveal items on lower zoom levels that were initially invisible. Either the visualization has several discrete hierarchy levels that are switched based on the zoom factor (such as the treemap example in Figure 6), or the visualization can be continuously zoomed (such as the HIV example in Figure 1). In the latter case, each hierarchy level has to be mapped onto an appropriate zoom factor, so that its associated items are optimally visible. Color blending assures that color transitions are seamless. To smoothen the transition between hierarchy levels in continuously zoomable visualizations (requirement 3), we linearly interpolate the colors in between the levels. The current hierarchy level is calculated by mapping the camera distance to discrete zoom levels. Currently, users have to manually define the mapping distance for each zoom level. Choosing the correct mapping distance parameters is important, otherwise significant issues can arise. For example, too many items may receive their own color, making them indistinguishable. In the future, we plan to investigate automatic camera‐distance settings, based on the screen size of the items associated with the zoom levels. From the camera distance, we are able to compute the current interpolation between two levels. The hue of an item is computed by performing a linear interpolation of the hue value of the two levels, as illustrated in Figure 13. In the use case of the HIV data set, the calculation is performed in a fragment shader. The different color components can be treated for each screen pixel individually. This way, information of multiple scales can be seen in a single visualization, as shown in Figure 14.

In the luminance‐modulated HIV example (Section 5.2), like the hue, the luminance value is also defined by the distance of the camera to the respective protein structures. However, given an interpolation factor derived from the zooming distance to the pre‐defined zoom levels, luminance values are interpolated between the next two hierarchy levels with the current interpolation factor, as illustrated in Figure 13. This way, hue and luminance encode different hierarchy levels and thereby generate an effect similar as shown in the close up in Figure 2. The seamless multi‐scale view‐guided color mapping transformation can be seen in the accompanying video S1.

## 7 Usability Study

**Figure 15:**
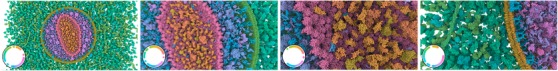
Various snapshots from the multi‐scale HIV visualization: As the camera position changes, the distinguishability between protein types is optimized. The color palette widget at the bottom left allows us to visualize the distribution of the groups along the hue ring. The hue ring is not intended to be directly interpreted. It is a technical complement to support intuitive understanding.

To showcase the capabilities of the Cuttlefish color mapping system, we conducted a usability study with the HIV model (Figure 15) using the coloring method from Section 4.2. Since interactively explorable multi‐scale visualizations of biology models have only become available very recently, there is no comparable approach how to represent biological structures across multiple scales. Dynamic visual discrimination, apart from geometric levels of detail [PJR*14], has not been studied so far in the biological field. We therefore decided for a qualitative evaluation since there is no clear baseline for a comparative laboratory experiment, in order to answer two research questions:

1. Does the dynamic color mapping support discrimination of protein structures on multiple scales?
2. Are the dynamic color changes distracting/unpleasant in the exploration process?

The participants are described in Section 7.1, tasks in Section 7.2 and results in Section 7.3.

### 7.1 Participants

To answer these two research questions, we asked five students and professionals in the field of molecular biology to perform two tasks. These five experts were: two PhD students, one postdoctoral fellow, one pharmacist and one master student; one female and four males; aged 24–31; all with normal or corrected‐to‐normal vision. The participants were given 20 Euros for participating. While dynamic color mapping is also aimed at the general audience, no such users were involved in the study. The task description for such users would have to be rather long and detailed, likely leading to issues with text comprehension or revealing too much information.

### 7.2 Tasks

There were two tasks in the usability study. In the first task (*structure identification task*), users were asked to identify structures on multiple levels of detail. In the second task (*free exploration task*), the users were told to freely browse through the model and think aloud.

**Figure 16:**
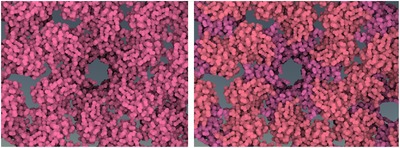
Comparison between static protein coloring (left) and hierarchical color coding using the Cuttlefish color mapping (right) for viewing a capsid protein hexamer on the protein domain level.

The Cuttlefish coloring scheme helps distinguish items. While this helps finding the items (Figure 16), as they are separated by color from the surrounding items, the exact identification requires knowledge of the structure itself. We based our task description on a previous publicly available description of the HIV capsid at PDB‐101 [Goo13]. Based on this description, users were asked to identify the following structures in the first task:

1. One of the 12 pentamer capsid proteins.
2. The N‐terminal and C‐terminal domain of the capsid protein.
3. The α‐helix in the N‐terminal domain stabilizing the hexamers/pentamers.
4. The binding site of cyclophilin A, which is a loop on the surface of the capsid protein with several proline amino acids.
5. One methionine amino acid within the α‐helix.

The last sub‐task was not included in the expert's task description, but was added as a representative task on the amino acid and atom level, respectively. Since we did not color‐code the amino acid level explicitly, users were given the hint to identify methionine based on the coloring of its sulphur atom. To assess the performance in the *structure identification task*, we recorded whether users were able to correctly identify the above listed structures.

In the subsequent *free exploration task*, users could freely navigate through the visualization, while thinking aloud. All reported insights were noted. Both tasks were recorded and followed by a questionnaire and a semi‐structured interview. Before the study, users could experiment with the tool to get familiar with the navigation, and were also instructed how to toggle the visibility of the protein groups.

With the structure identification task, we could assess whether experts with sufficient knowledge to understand molecular structures without additional text labels are able to identify the above structures using our system. While Cuttlefish provided the necessary discrimination of individual structures in the respective zoom levels, the identification of the structures was only possible through their structural properties. The only exception was the methionine amino acid, which was indicated by the yellow sulphur atom. Through the free exploration, our goal was to assess whether users would notice and be distracted by our dynamic color mapping.

As mentioned, structural information alone is not sufficient to discriminate structures below the protein level, as shown in Figure 16. In addition, performance measures of complex tasks are rather hard to compare with only a small number of expert users executing the study.

### 7.3 Results

Table 1 summarizes the performance of users in the structure identification task. Except for user 3 (the pharmacist), all users were able to correctly identify all structures down to the secondary structure level. User 3 mixed up the N‐ and C‐terminals of the capsid protein, and the rest of the tasks are to some degree based on finding them. With regard to the amino acid, there was more than one kind of amino acid with a sulphur atom, and two users mistook this amino acid for the correct one.

**Table 1:** Structure identification task: **c** identified correct structure, **p** partially identified correct structure, **i** identified incorrect structure, **n** nothing found

Task \user	1	2	3	4	5
Pentamer	**c** (5)	**c** (5)	**i** (4)	**c** (5)	**c** (5)
C‐terminal	**c** (5)	**c** (4)	**i** (1)	**c** (4)	**c** (4)
N‐terminal	**c** (5)	**c** (4)	**i** (1)	**c** (2)	**c** (4)
α‐helix	**c** (1)	**c** (3)	**n**	**c** (4)	**c** (2)
Binding site	**c** (1)	**c** (5)	**c** (5)	**c** (5)	**c** (4)
Methionine	**c** (1)	**p** (5)	**n**	**p** (1)	**n**

User‐reported certainty shown on a scale of 1 (lowest) to 5 (highest).

In the questionnaire, users assessed the identification of compartments, but also of proteins, as very easy (Table 2). However, the domain and secondary structure identification was rated as much more difficult. This is also reflected in the users' self‐reported certainty of the identified structures (Table 1). While the users were quite certain about the identity of a pentamer capsid, once they spotted it, they were least certain about the α‐helices stabilizing the hexamers and pentamers. All users, except for user 3, were able to identify the C‐ and N‐terminal domains of the capsid proteins. All those users verbally referred to the domains by color. Two users also referred to the α‐helices by color.

**Table 2:** User answers on a five‐point Likert scale for questions concerning the ease of identification of structures (top five), as well as color changes and visual appeal (bottom three)

Question	Average	All values
Compartment	4.8	5, 4, 5, 5, 5
Capsid proteins	4.4	5, 4, 3, 5, 5
Protein domains	3	2, 4, 2, 3, 4
Secondary structures	2.2	1, 2, 2, 2, 4
Atomic structure	3	3, 2, 3, 2, 5
Notice color change	4	5, 5, 1, 4, 5
Color change confusion	2	1, 1, 3, 3, 2
Visual appeal	5	5, 5, 5, 5, 5

In the free exploration task, all except for user 3 explored the virus. On average, they spent 10 min on the exploration. Some users reported that they learned something new when exploring the visualization, such as that *‘HIV uses [the] host membrane'* and *‘how crowded everything is'*. The four users focused on different parts of the virus during their free exploration, such as the membrane (users 1 and 5), the proteins in the matrix (users 1 and 4) and the amino acids of the cyclophilin A binding site (user 2).

In the questionnaire, most users indicated that they noted the color changes (Table 2), but the confusion caused by these color changes was rated to be fairly low. When asked about the changing colors in the post‐experiment interview, all users reported that they noticed changing colors when zooming to the atomic level. However, only one user noticed it on all levels, while a second one thought *‘something odd'* was occurring on the secondary structure level.

In general, all users issued the highest possible grade for visual appeal in our questionnaire. In the post‐experiment interview, they explained that they considered the tool useful for presenting research and educating students – which is in line with our research goals. They also had suggestions for improvement, such as adding text labels, visually marking the termini of the protein domains and providing a cartoon representation for secondary structures.

### 7.4 Summary and discussion of results

The performance of and feedback from the molecular biologists in our study indicate that the dynamic color mapping supports users in identifying molecular structures on multiple scales. Users were equally successful in identifying one of the capsid proteins forming a pentamer, and in identifying the two domains of the capsid protein. The pentamer capsid protein differed only in shape from the more frequent hexamer capsid proteins, while the two different domains of the capsid proteins were encoded by color. The users reported lower certainty and higher task difficulty for the identification of the domains. This is an indication that identification by structure is easier than identification by color alone – yet, color can be used if no strong structural cues are available, as in our protein domain example.

On the secondary structures level, the identification rate was high, but users were quite uncertain about their findings and reported a high task difficulty. On this level, α‐helices and β‐sheets were distinguished by color, similarly to Figure 12. However, the particular α‐helix mentioned in the task description could only be identified as a helix by shape. The request for an alternative cartoon representation for identifying structures on this level shows the limits of multi‐scale color mapping without adapting the structural representation. In the future, it will be important to explore combinations of semantic zooming comprising both structural and color information for multi‐scale biological visualizations.

User feedback shows that our dynamic color changes do not interfere with the users’ workflow. While protein domains and secondary structures were mainly referred to by their color, only few users noticed the color changes (except at the atomic level) and none found them confusing. All users found the visualization highly appealing and useful for presentation and education purposes. We therefore conclude that the Cuttlefish color mapping for dynamic multi‐scale visualizations is a valuable extension to multi‐scale molecular visualization. It does not cause any notable distraction and allows for an efficient and pleasing visual encoding of protein substructures.

The usability study was designed to test the usefulness of the hue change. The coloring method was the one used in Section 4.3. The luminance modulation was not added for two reasons. First, we did not ask the user to find anything based on quantity, so the modulation according to Section 5.1 would not have been of any use. Second, we asked the users to find an item on a specific level, and they were not required to compare two levels at once. Therefore, the luminance modulation for substructures (Section 5.2) was not applied here.

## 8 Conclusion

The Cuttlefish color mapping technique can color a hierarchical data structure coherently and cohesively over multiple levels. The hierarchical structure of the coloring technique, along with the visibility based variation in color, allows users to navigate and inspect parts of the scene without getting disorientated while only introducing subtle color changes. Altering the color based on semantic zooming changes the meaning of color coding. Instead of distinguishability for any situation, semantic color changes allow items to be clustered or separated as necessary. This is achieved while maintaining the connection between groups and items on different levels in a logical manner via the hierarchical nature of the coloring. Furthermore, the coloring scheme is capable of showing items on each level in a visually distinct manner. The usability study was performed with a research prototype and shows that experts can find information in an HIV model on each level, while not being distracted by the dynamic color mapping. This means that, while the coloring method may seem unnatural due to the potentially significant change in color from one level to another, it does not seem to be a cause for concern.

There are still open questions. First, our method helps distinguishing items. This is very basic, and supporting a more complex task, such as identification, would be useful. Second, the method currently relies on the user setting the distances between objects and camera for the different levels manually. Third, we use a simple hue wheel as a basis. The use of coloring methods based on alternatives to analogous hue wheels has not yet been investigated. However, Cuttlefish is a fast, easy to implement method that can be used on any hierarchical data set.

## Supporting information


**Video S1**
